# Development of an algorithm to identify fall-related injuries and costs in Medicare data

**DOI:** 10.1186/s40621-015-0066-z

**Published:** 2016-01-05

**Authors:** Sung-Bou Kim, David S. Zingmond, Emmett B. Keeler, Lee A. Jennings, Neil S. Wenger, David B. Reuben, David A. Ganz

**Affiliations:** 1 Pardee RAND Graduate School, 1776 Main Street, Santa Monica, CA 90407 USA; 2 Division of General Internal Medicine and Health Services Research, David Geffen School of Medicine at UCLA, Center for Health Sciences, Los Angeles, CA 90095 USA; 3 RAND Health, RAND Corporation, 1776 Main Street, Santa Monica, CA 90407 USA; 4 Multicampus Program in Geriatric Medicine and Gerontology, David Geffen School of Medicine at UCLA, Center for Health Sciences, Los Angeles, CA 90095 USA; 5 Geriatric Research, Education and Clinical Center, and Center for the Study of Healthcare Innovation, Implementation and Policy, Veterans Affairs Greater Los Angeles Healthcare System, 11301 Wilshire Blvd (11G), Los Angeles, CA 90073 USA

**Keywords:** Fall, Fall-related injury, Algorithm, Medicare, ICD-9-CM, HCPCS

## Abstract

**Background:**

Identifying fall-related injuries and costs using healthcare claims data is cost-effective and easier to implement than using medical records or patient self-report to track falls. We developed a comprehensive four-step algorithm for identifying episodes of care for fall-related injuries and associated costs, using fee-for-service Medicare and Medicare Advantage health plan claims data for 2,011 patients from 5 medical groups between 2005 and 2009.

**Methods:**

First, as a preparatory step, we identified care received in acute inpatient and skilled nursing facility settings, in addition to emergency department visits. Second, based on diagnosis and procedure codes, we identified all fall-related claim records. Third, with these records, we identified six types of encounters for fall-related injuries, with different levels of injury and care. In the final step, we used these encounters to identify episodes of care for fall-related injuries.

**Results:**

To illustrate the algorithm, we present a representative example of a fall episode and examine descriptive statistics of injuries and costs for such episodes. Altogether, we found that the results support the use of our algorithm for identifying episodes of care for fall-related injuries. When we decomposed an episode, we found that the details present a realistic and coherent story of fall-related injuries and healthcare services. Variation of episode characteristics across medical groups supported the use of a complex algorithm approach, and descriptive statistics on the proportion, duration, and cost of episodes by healthcare services and injuries verified that our results are consistent with other studies.

**Conclusions:**

This algorithm can be used to identify and analyze various types of fall-related outcomes including episodes of care, injuries, and associated costs. Furthermore, the algorithm can be applied and adopted in other fall-related studies with relative ease.

**Electronic supplementary material:**

The online version of this article (doi:10.1186/s40621-015-0066-z) contains supplementary material, which is available to authorized users.

## Background

Falls and fall-related injuries are costly to society, and most commonly occur in the older population (Tinetti et al. 2008). Although the impact of falls on costs and interventions to reduce fall-related injuries are major areas of ongoing research, identifying fall-related injuries poses a major challenge for researchers since tracking actual falls requires examining medical records or patient self-report augmented with memory aids, such as calendars or diaries (Ray et al. 1992; Rizzo et al. 1996; Rizzo et al. 1998). Medical record review is expensive and self-report may be inaccurate because of recall problems or the hassle of completing memory aids (Ganz et al. 2005). The use of healthcare claims data provides an alternative way of identifying fall-related injuries that is cost-efficient and easier to implement (Bohl et al. 2010; Carter and Porell 2011; Finkelstein et al. 2005; Tinetti et al. 2008). Furthermore, the accuracy of identifying injuries from claims may increase when using complex algorithms instead of simple definitions (Ray et al. 1992).

In addition to their potential utility in capturing fall-related injuries, healthcare claims may be helpful more generally in testing different definitions of episodes of care for purposes of refining alternative approaches to reimbursement, such as bundled payment. The concept of an episode of care has long been the subject of public interest and debate for reforming the healthcare system. From the Medicare Participating Heart Bypass Center Demonstration in 1991 to the recent Bundled Payments for Care Initiative in 2013, efforts have been made to explore episode-based bundled payment systems that would enhance coordination among providers, improve the quality of care, and reduce healthcare expenditures (Chen et al. 2015; Mechanic 2015). Moreover, the Centers for Medicare and Medicaid Services (CMS) has proposed the Comprehensive Care for Joint Replacement program to begin in January 1, 2016, which is a mandatory Medicare bundled payment scheme for hip and knee replacements (Chen et al. 2015; Mechanic 2015). In this study, we build on previous work to develop a novel approach to identifying episodes of care for fall-related injuries.

Empirical studies have used various measures and types of information to define and classify falls or fall-related injuries. Some studies have classified falls or fall-related injuries based on the setting in which injuries are treated, such as acute care hospital or emergency department (ED) visits (Bohl et al. 2010; Finkelstein et al. 2005; Stevens et al. 2006). Others have used more sophisticated definitions that also involve body sites or types of injury such as hip and other fractures, head injuries, and joint dislocations (Ray et al. 1992; Rizzo et al. 1996; Rizzo et al. 1998; Tinetti et al. 2008). Recent studies have incorporated both severity and body location to identify falls and injuries (Bohl et al. 2010; Bohl et al. 2012; Carter and Porell 2011). For example, Carter and Porell (2011) use location as a measure of injury severity to identify hospitalized and non-hospitalized fallers while Bohl et al. (2012) assign an Injury Severity Score (ISS) to the identified injury episodes and use this information to distinguish sentinel injuries from minor injuries.

Real-life evaluations of falls prevention include a complex mix of patients moving between payers, even within Medicare. However, most studies using administrative data have examined claims data from either traditional fee-for-service Medicare, or health maintenance organizations, but not both due to the complexity involved in obtaining and combining the datasets. Studies have typically used general International Classification of Diseases, Ninth Revision, Clinical Modification (ICD-9-CM) diagnosis codes as well as fall-related external cause of injury codes (E codes) to identify fall-related claims (Bohl et al. 2010; Ray et al. 1992; Tinetti et al. 2008). E codes can be used to identify fall-related claims where their use is mandated, a state-by-state decision. However, in studies that span several states, reliance on E codes may result in incomplete identification of fall-related injuries and potential misclassification of injured and non-injured individuals.

The outcome of interest also affects how falls or fall-related injuries are defined. For example, specific fractures for each body site can be counted so that a single injury event could involve multiple fractures (Ray et al. 1992). Counts of injuries are often used to calculate occurrence rates (e.g., per 1000 person-years) (Tinetti et al. 2008). Another approach is injury episodes, which include all injuries within a designated window of days (e.g., 90 or 180 days) (Carter and Porell 2011). While some earlier studies used charge data, more recent studies used medical expenditures as a close estimate of fall-related costs (Alexander et al. 1992; Bohl et al. 2010; Carter and Porell 2011; Finkelstein et al. 2005; Rizzo et al. 1998).

However, studies have rarely taken all of these factors into account when using claims data to identify fall-related injuries. And while each study estimates the impact of falls and fall-related injuries, it is difficult to compare the results across studies since they involve different definition of falls, different information, and different assumptions. Therefore, we developed a comprehensive algorithm for identifying episodes of care for fall-related injuries, which we defined as a collection of fall-related injury claims that are clustered closely enough within a specified time period to believably stem from the same underlying injury. In this algorithm, we identified fall-related injuries by their healthcare services setting, body sites, and by types of injury. We also used both fee-for-service (FFS) Medicare data from CMS and Medicare Advantage (MA) health plan data, which required integrating datasets from different sources with variation in data format and availability. (For simplicity, we use the term “claims” to refer to data from both FFS Medicare and MA health plans, even though not all MA data are generated from requests for payment.) In particular, the algorithm involved the use of E codes (when available) as well as general ICD-9-CM diagnosis codes, and identified fall-related injuries as well as episodes of care so that both frequencies and costs of fall-related injuries could be examined in detail.

Our algorithm builds on previous work in several ways. We used work  by Taylor et al. and Ray et al. that applied complex algorithms to Medicare data (specifically, inpatient, ED, outpatient, and physician files) to examine incidence of fractures (Ray et al. 1992; Taylor et al. 2011). We adopted the classification of the five types of injuries (hip fractures, other fractures, head injuries, joint dislocations, and fall-related use of medical services (E codes)) from Tinetti et al (2008). We follow Bohl et al. (2012) and also use location as a proxy for severity of the injuries. Our algorithm provides two particularly novel contributions to the field. First, the algorithm is flexible with regard to the sources of claims data; specifically, the algorithm can make use of both FFS Medicare and MA health plan data together. Second, the definition of an episode is more refined, since it is based on the actual pattern of claims rather than a fixed time window, thereby allowing actual care rendered to drive episode duration and thereby capture the appropriate costs.

## Methods

We developed an algorithm to analyze fall-related outcomes for the ACOVEprime study, which involved a multicomponent intervention in primary care practices to improve the quality of care for falls (Ganz et al. 2015; Wenger et al. 2010). We limited our data analysis to a group of patients age 75 and older with high risk of falling, who were receiving care from practices located in five distinct locations, and covered by either FFS Medicare or MA. We collected Medicare enrollment information and claims data for 2,011 patients from 5 medical groups between 2005 and 2009. We obtained Medicare FFS data, as well as comparable MA datasets from 5 health plans in 2 medical groups (We provide the descriptions of the datasets, data cleaning and combining process, and key variables used in the algorithm in Additional file 1: Appendix A since the information is important for understanding the results as well as for applying the algorithm in a different context).

Using merged Medicare FFS and MA datasets, we developed an algorithm which involved four steps to identify episodes of care for fall-related injuries and associated costs. We describe the algorithm in stepwise fashion to illustrate how we coded and executed the algorithm. First, as a preparatory step, we identified events with care received in acute inpatient and skilled nursing facility (SNF) settings, in addition to ED visits. Second, we identified all records with diagnosis and procedure codes relevant to falls or fall-related fractures. Third, with these records, we defined and identified six different types of encounters for fall-related injuries, based on the healthcare setting and type of codes involved. Fourth, based on these encounters, we identified episodes of care for fall-related injuries.

The first step involved preparatory work for later steps of the algorithm. First, we identified claims and line items for care provided in the inpatient and SNF setting; this information was used in the third step of the algorithm. We identified inpatient and SNF claims for FFS datasets using the short stay/long stay/SNF indicator code variable included in the MedPAR dataset. For MA datasets, which varied in the type and availability of information, we used a case-by-case approach utilizing all available information (see Additional file 1: Appendix B). For both FFS and MA datasets, we also applied a list of Current Procedural Terminology (CPT-4) codes (also known as HCPCS level I codes) that identified inpatient and SNF settings; for identifying the inpatient setting, we used codes 99217–99223, 99231–99236, 99238–99239, 99251–99255, 99261–99263, 99291–99292, and 99356–99357 whereas for the SNF setting we used codes 99301–99313, 99315–99316, and 99318.

Another process in step 1 involved identifying ED visits, independent of inpatient visits, which was used in the fourth step of the algorithm. A claim or line item was considered as an ED visit or related activity if it satisfied one of the following three criteria: (1) Berenson-Eggers Type of Service (BETOS) code, which is based on the HCPCS code, is “M3” or starts with the alphabet letter “I”, (2) place of service code refers to “Emergency Room – Hospital”, or (3) revenue code is 450–459 or 981 (Centers for Medicare & Medicaid Services; Merriman and Caldwell 2003).

The second step of the algorithm involved identifying all fall-related incidents using claims and line items with diagnosis and procedure codes relevant to fractures or fall-related injuries. For diagnosis codes we used all available primary and secondary ICD-9-CM codes, including E codes. For procedure codes we used CPT-4 codes which we further distinguished as repair, casting, splinting, or imaging codes. All fall-related incidents were classified as one of the following five types of injuries: hip fracture, other fracture, head injury, joint dislocation, or E codes for accidental falls. Additional file 1: Appendix C provides a detailed description of the different types of injury and associated diagnosis and procedure codes used.

**Fig. 1:**
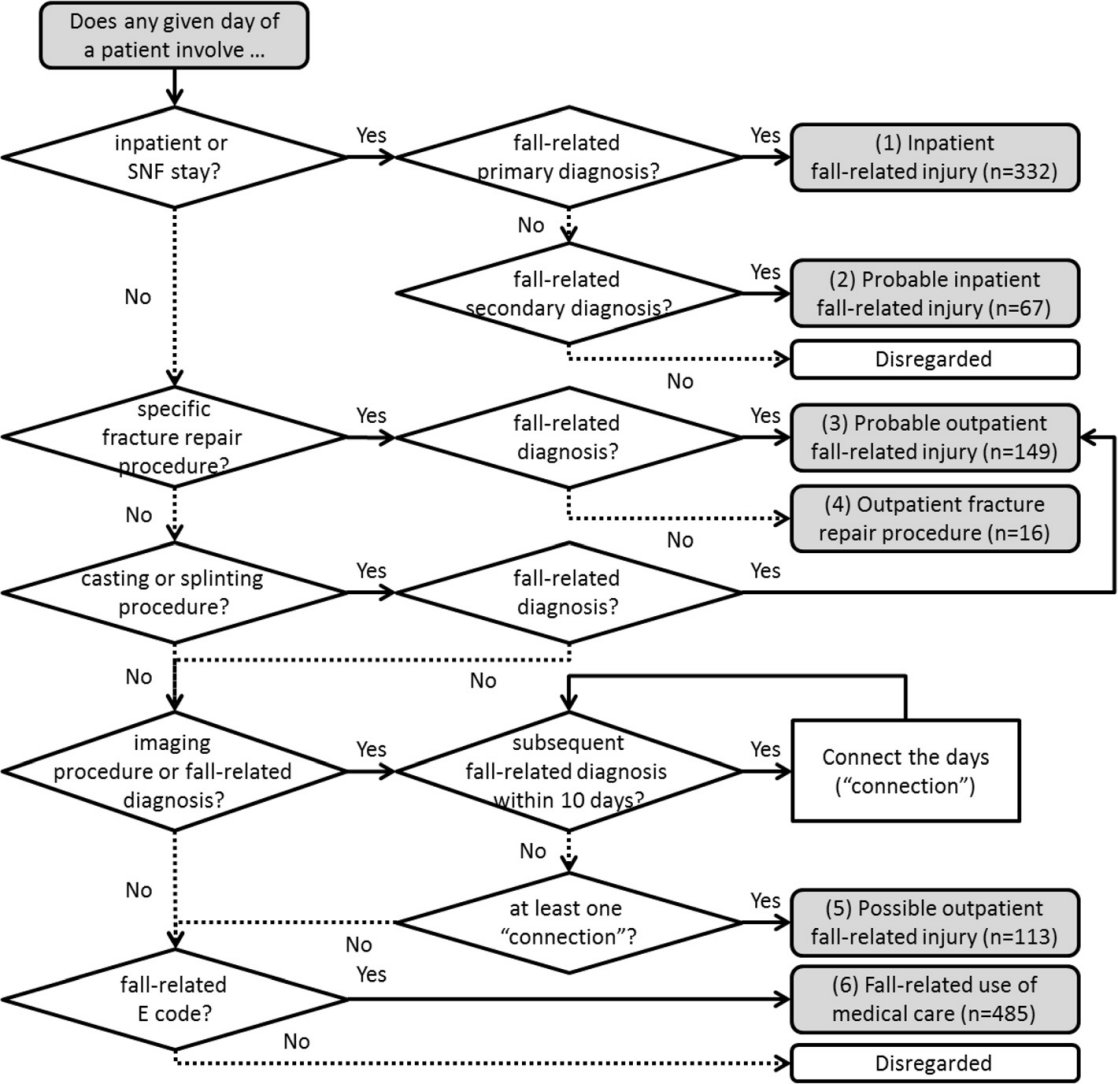
Flow diagram for the third step of the algorithm. The flow diagram above consists of three different types of boxes and two types of arrows. Rounded rectangles represent start or end, rectangles represent processes, and diamonds represent decisions. When used alone, solid arrows represent the direction of flow. When both solid and dotted arrows originate from a decision box, they each represent responses of “yes” and “no”, respectively. For each iteration, start on the top left corner of the figure, at the shaded rounded rectangle with the outbound solid arrow, and end in one of the eight rounded rectangles on the far right. Six shaded rectangles refer to the different types of encounters while the other two refer to invalid cases. For each type of encounter, the final number of encounters is included in parenthesis

The third step of the algorithm involved classifying each day of fall-related care into six different types, as shown in Fig. 1; we refer to these types as ‘encounters’ for fall-related injuries. The different types of encounters are in hierarchical order, with lower (better-ranked) numbers representing higher certainty that the encounter involves fall-related injuries and procedures for such injuries. (Hereafter, we will use numbers 1 to 6 in parenthesis to refer to the six types of encounters.) First, we determined whether a given day of a patient involved an inpatient or SNF stay. If it did, and also involved a fall-related diagnosis on the same day, then we considered it as an inpatient fall-related injury encounter. Depending on whether the fall-related diagnosis is primary or secondary, we classified it as an “(1) inpatient fall-related injury” or a “(2) probable inpatient fall-related injury”, respectively. For a given day that did not involve an inpatient or SNF stay, we proceeded as follows. If we found a repair procedure that was specific to a body site or type of injury, then we considered it as a fall-related injury encounter. Depending on whether there was also a fall-related diagnosis or not, we classified the encounter as a “(3) probable outpatient fall-related injury” or an “(4) outpatient fracture repair procedure”, respectively. If there was a general casting or splinting code on a given day, accompanied by a fall-related diagnosis for which a cast or splint would be appropriate (see Additional file 1: Appendix C), then we also classified it as a “(3) probable outpatient fall-related injury”.

If a given day did not involve an inpatient or SNF stay and did not qualify as a “(3) probable outpatient fall-related injury” or an “(4) outpatient fracture repair procedure”, we applied the following process. If a given day involved a fall-related diagnosis or an imaging procedure, we looked forward up to 10 days after the given day and searched for another day with a diagnosis code on the same body site or type of injury (as listed in Table C.1 in Additional file 1: Appendix C). If so, we “connected” (i.e., linked) the days of the two sets of records. Using the latter day as the reference date, we repeated this search and connection process until we no longer observed a subsequent day with a relevant diagnosis code within a 10 day window. If at least one connection occurred for a given day, we considered all connected days as a single “(5) possible outpatient fall-related injury.” For patient days that were not identified as any of the encounter types above, we examined whether they involved an E code for accidental falls. If so, we classified these days as “(6) fall-related use of medical care.”

We repeated this process until all fall-related incident days were examined and fall-related injury encounters were identified, where present, for all patients. Based on clinical judgment, we applied the process at differing levels for each of the five types of fall-related injury (that were identified in the second step of the algorithm). Specifically, for “hip fracture” and “other fracture”, we identified encounters (1) to (4). For “other fracture” with associated list of imaging codes and “joint dislocation”, we identified encounters (1) to (5). For “head injury,” we only examined these codes in the inpatient and SNF setting and identified encounters (1) and (2). For “fall-related E codes”, we applied all steps of the flow diagram and identified encounters (1) to (6).

**Fig. 2:**
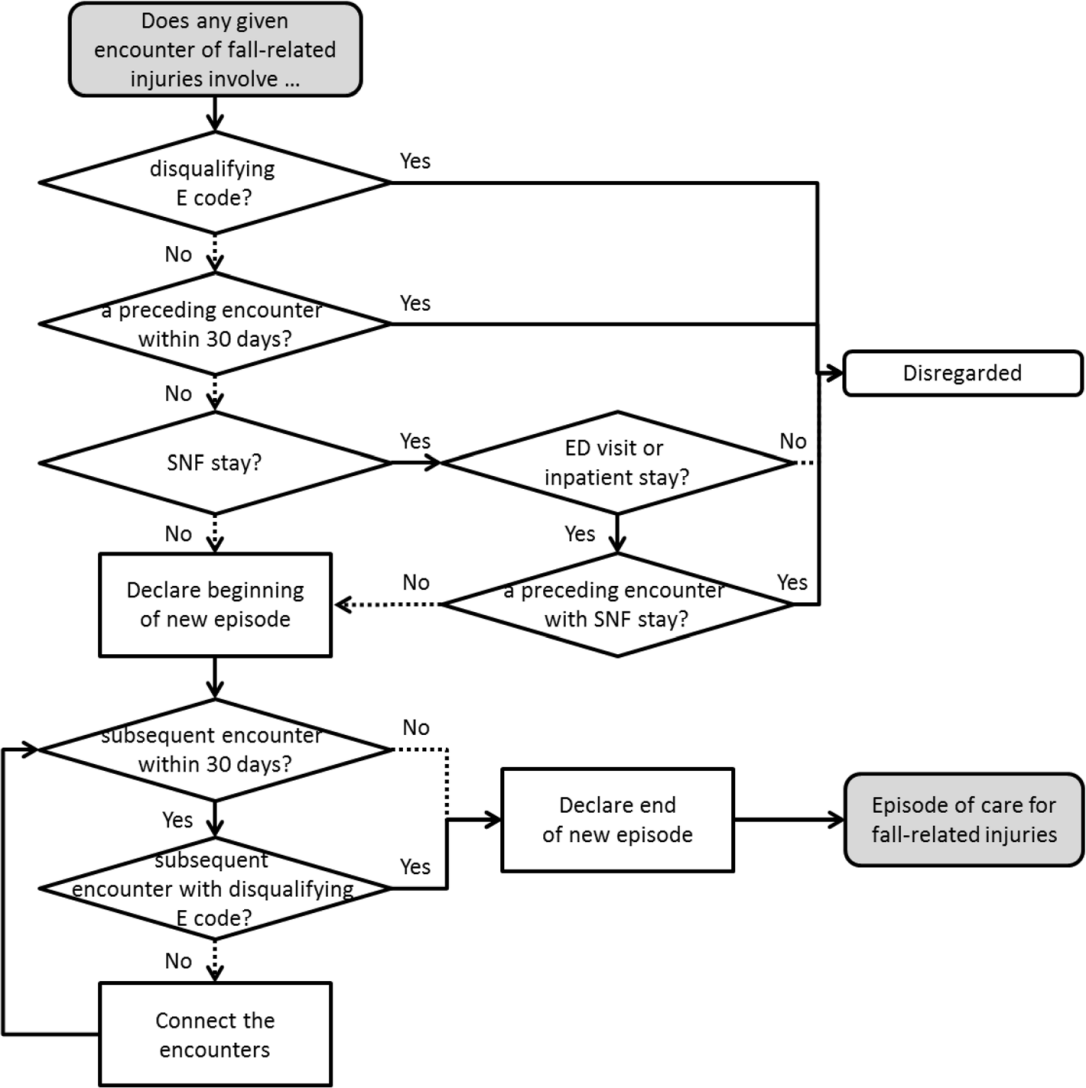
Flow diagram for the fourth step of the algorithm. See Fig. 1 for descriptions of the different types of boxes and arrows. For each iteration, start on the top left corner of the figure, at the shaded rounded rectangle with the outbound solid arrow, and end in one of the two rounded rectangles on the far right. The shaded rounded rectangle refers to the identification of a new episode of care for fall-related injuries, while the other refers to invalid cases

The fourth and final step involved identifying episodes of care for fall-related injuries, as shown in Fig. 2. The initial part of the diagram consists of steps to identify the beginning of a new episode of care for fall-related injuries. First, we confirmed that a given encounter did not involve any disqualifying E codes (i.e., E codes suggesting a mechanism of injury incompatible with falls as a primary cause; see Table C.2 in Additional file 1: Appendix C). Then we looked back 30 days from the beginning date of the encounter to verify that there were no preceding encounters of any type, to confirm that the encounter in question was not potentially part of an earlier episode of care. If there were no preceding encounters, we examined whether the given encounter involved a SNF stay. If it did not involve a SNF stay, we identified this encounter as the beginning of a new episode. If it did involve a SNF stay, we applied additional requirements that the (i) given encounter must also involve an ED visit or inpatient stay and the (ii) preceding encounter should not involve a SNF stay. With these requirements, we ensured that this encounter indeed qualified as the beginning of a new episode with valid entry into SNF stay.

Then, we focused on identifying the end of this episode. Since an episode of care may involve injuries at several body sites at different points of time with short intervals, we first examined whether there was any other subsequent encounter within 30 days of the given encounter (that represented the beginning of a new episode). If there was a subsequent encounter within 30 days, and this encounter did not involve a disqualifying E code, then we connected these encounters together. We repeated this process until we were unable to find any subsequent encounter within 30 days of the latest connected encounter. We also stopped the process if we came across a subsequent encounter with a disqualifying E code. Once this process came to an end, we defined the latest connected encounter as the end of the episode, and defined the connected set of encounters as a new episode of care. We repeated the whole process until all encounters were examined and episodes of care for fall-related injuries were identified, where present, for all patients.

We also sorted each episode into six mutually exclusive and hierarchical categories, which was based on the most severe and reliable level of injury and care involved in each episode. For example, an episode with “(1) inpatient fall-related injury” and “(4) outpatient fracture repair procedure” encounters was considered as an episode of care involving “(1) inpatient fall-related injury.”

Subsequently, we estimated the cost of healthcare services for each episode, which was adjusted to 2009 U.S. dollars using the medical care component of the consumer price index for all urban consumers (Bureau of Labor Statistics). We calculated two types of cost, namely total and attributable cost. Total cost involved all costs during the duration of each episode of care, while attributable cost only included cost of claims for healthcare that had a fall-related diagnosis code.

## Results and discussion

First, we present a representative example of an episode of care for fall-related injuries to illustrate way in which an episode of care is identified using the algorithm. Then we examine descriptive statistics of episodes including frequency, duration, and cost, which corroborate that the algorithm is functioning as intended.

### Representative example of an episode of care

**Fig. 3:**
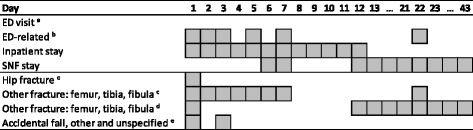
Representative example of an episode of care for fall-related injuries. ^a^ Beneficiaries seen in ED are identified by revenue center code values of 0450–0459 and 0981. ^b^ Other charges possibly associated with emergency rooms are identified by place of service code and BETOS code. ^c^ Primary diagnosis code. ^d^ Secondary diagnosis code. ^e^ External cause of injury code

Inpatient stays involving hip fractures were most commonly observed among the episodes of care (201/1162 = 17 %), and average length for these episodes was approximately 43 days. In Fig. 3, we present a real episode of a FFS patient lasting 43 days that involves an inpatient stay with hip fracture. The columns represent the days counting from the beginning to the end of the episode. The first four rows show the healthcare setting from which claims were filed during this period. Revenue center codes did not confirm the presence of an ED visit for this episode since ED visits that result in hospitalization are not billed separately. However, ED-related costs (involving chest and musculoskeletal X-rays) were observed at the earlier stage of the episode, which confirmed the presence of ED visits. Also, based on the start and end of service dates for the filed claims, we found that the episode involved an inpatient stay from the first day up to the sixth day, and then the patient was transferred to and treated at SNF. However, this patient returned to inpatient setting the next day and stayed there for nearly a week, and then was relocated to SNF until the end of this episode.

The bottom four rows of Fig. 3 show the body sites and types of injuries. We see that this episode involved injuries at more than one body site as it involved a primary diagnosis of hip and other (specifically femur, tibia, or fibula) fractures at the beginning of the episode. Although we cannot determine the exact mechanism of injury for the accidental fall, being listed as “other and unspecified,” the presence of fall-related E codes (E888.9) supports our algorithm’s classification of this episode as being fall-related.

Inpatient and SNF stays accounted for 93 % ($ 38,854/$ 41,963) of all costs during this episode, and almost two thirds of all costs were accrued in the first twelve days of the episode, consistent with the clinical expectation that acuity of care is highest in the early phase of an episode. Examination of the remaining claims data for this patient revealed an additional episode of care, 4 months prior to this episode, which involved a same level tripping code for accidental fall (E885.9) and a single-day outpatient visit with radiologic examination of the shoulder. We did not find any fall-related claims in between or after these episodes.

### Descriptive statistics for episodes of care

Our descriptive analyses began by examining how different types of healthcare settings and injuries were associated with episodes of care by medical group. We observed a wide variation in outcomes across medical groups. The columns in Table 1 represent different medical groups. The first two rows represent the raw number of patients and episodes by medical group while the third row provides standardized episodes per 1000 person-years. The mean number of standardized episodes per 1000 person-years was 144, while displaying variation across medical groups from the lowest at 88 to the highest at 189.

**Table 1:** Episodes of care by types of healthcare services setting and injuries involved

	Medical group					
	A	B	C	D	E	Total
Total patients	1018	304	276	350	63	2011
Total episodes	676	209	105	130	42	1162
Standardized episodes (per 1000 person-years) ^a^	156	189	121	88	147	144
Proportion of episodes, by healthcare services ^b^						
Inpatient or SNF stay (Inp. / SNF)	39 %	52 %	36 %	49 %	43 %	43 %
ED visit or ED-related visit (ED)	52 %	41 %	54 %	42 %	55 %	49 %
Non-ED outpatient visit only (Out.)	9 %	7 %	10 %	9 %	2 %	8 %
Proportion of episodes, by injury ^b^						
Hip fracture	17 %	20 %	19 %	16 %	19 %	18 %
Non-hip fracture	32 %	39 %	30 %	52 %	33 %	35 %
Non-fracture injury	51 %	41 %	51 %	32 %	48 %	47 %

^a^ Standardized episodes refer to number of episodes per 1000 person-years, since total episodes do not reflect the difference in person-years among medical groups

^b^ These rows are hierarchical and mutually exclusive, with the highest level of care experienced in the category coming first

The next four rows display the proportion of episodes involving different types of healthcare services. All episodes involving an inpatient or SNF stay are counted in the fifth row. Episodes that do not involve inpatient or SNF stay but involve an ED visit fall into the sixth row. The rest of the episodes are categorized as episodes with “non-ED outpatient visit only.” A significant proportion of episodes of care did not involve an inpatient or SNF stay (mean of 57 %, ranging from 48 % to 64 % by medical group).

The last four rows are also mutually exclusive categories that show the proportion of episodes involving different types of injuries. Any episodes that involve hip fractures are categorized as “hip fracture.” Episodes that do not involve hip fractures are further categorized by whether they involve any other fractures or not, and are presented in the last two rows, respectively. Here, we see that a significant proportion of episodes of care were non-fracture injuries, with a mean of 47 %, and ranging from 32 % to 51 % by medical group.

Next, we examined body sites and injuries for episodes of care by type of healthcare services. The first column in Table 2 shows the number of episodes by body sites and type of injury, which are not mutually exclusive. The next three columns each show the proportion of episodes that involve inpatient or SNF stay, ED visit, or non-ED outpatient visit only. We find that the combined proportion with ED or outpatient visit is comparable with the results from Ray et al., which are shown in the last column (Ray et al. 1992). The comparability with Ray and colleagues’ results is reassuring, since their study used Medicare claims data to identify fractures and also compared a sample of identified fractures with medical records. Additionally, we see that a significant proportion of episodes in the outpatient setting involve ED visits (49 %/(49 % + 8 %) = 86 %), which suggests that the algorithm is appropriately identifying episodes of care of sufficient severity to be of interest to researchers and policymakers.

**Table 2:** Episodes of care by body sites, injury, and types of healthcare services

	No. of	Proportion with…			Ray et al.
Body sites and causes of injury ^a^	Episode	Inp. / SNF	ED	Out.	ED & Out. ^b^
Hip fracture	206	98 %	2 %	0 %	5 %
Other Fracture : Pelvis	53	85 %	15 %	0 %	14 %
Other Fracture : Rib	61	57 %	41 %	2 %	48 %^c^
Other Fracture : Clavicle	64	16 %	55 %	30 %	62 %^d^
Other Fracture : Humerus	91	51 %	44 %	5 %	55 %
Other Fracture : Radius & ulna	104	30 %	61 %	10 %	72 %
Other Fracture : Navicular (scaphoid)	34	41 %	53 %	6 %	
Other Fracture : Hand	44	18 %	80 %	2 %	86 %
Other Fracture : Femur, tibia, fibula	93	88 %	11 %	1 %	15 %^e^
Other Fracture : Patella	11	73 %	27 %	0 %	43 %
Other Fracture : Ankle	32	47 %	44 %	9 %	55 %
Head Injury : Head fracture	49	35 %	49 %	16 %	55 %
Head Injury : Head trauma	68	87 %	12 %	1 %	
Joint Dislocation : Shoulder	18	50 %	44 %	6 %	
Joint Dislocation : Elbow	0				
Joint Dislocation : Wrist	4	50 %	50 %	0 %	
Joint Dislocation : Knee	27	15 %	44 %	41 %	
E Codes : Stairs or steps	20	25 %	70 %	5 %	
E Codes : Ladders or scaffolding	1	0 %	100 %	0 %	
E Codes : Building or structure	0				
E Codes : Hole	0				
E Codes : One level to another	73	37 %	62 %	1 %	
E Codes : Same level - tripping	211	39 %	56 %	4 %	
E Codes : Same level - pushed	3	67 %	33 %	0 %	
E Codes : Other and unspecified	589	42 %	53 %	6 %	
All	1162	43 %	49 %	8 %	37 %

^a^ Body sites and causes of injury are based on ICD-9-CM diagnosis codes and external cause of injury codes, respectively (see Additional file 1: Appendix C)

^b^ Values in this column are cited from Table 3 in Ray et al.(Ray et al. 1992) which did not distinguish outpatient fractures by whether they involved ED visits or not. These proportions were based on a total of 2,398 probable fractures

^c^ Ray et al. refers to rib/sternum

^d^ Ray et al. refers to clavicle/scapula

^e^ Ray et al. identifies 15 % of femoral shaft fractures and 43 % of tibia/fibula fractures from outpatient claims

Lastly, we examined the duration and cost of episodes, in total and stratified by type of healthcare services and injuries. We found that the costs involved in episodes of care were comparable to other actual and predicted costs of fall-related injuries in the literature (Bohl et al. 2010; Finkelstein et al. 2005; Rizzo et al. 1998). The first column in Table 3 shows the mean duration of episodes of care while the next three columns each show the mean number of days for ED visits, inpatient, and SNF stay, respectively. Total and attributable costs are also shown in the last two columns of the table. (Arguably, we can assume that these two types of costs provide an upper and lower bound for the true cost of fall-related injuries, respectively.) As expected, episodes that involved inpatient or SNF stay were significantly longer and are more costly than episodes that did not, regardless of whether they involved an ED visit. In fact, the duration of non-inpatient/non-SNF episodes did not differ much between episodes with ED visits and episodes in the non-ED outpatient setting only. We also found that episodes with different types of injuries varied even more in terms of their duration and cost. The episodes with hip fractures showed longer duration and higher cost than episodes with non-hip fractures, as expected; similarly, episodes of other (non-hip) fractures were longer duration and higher cost than non-fracture episodes. Despite the relatively low cost involved in non-inpatient/non-SNF and non-fracture episodes, it was important that we also identify these episodes since any kind of injury can lead to undesirable health consequences in the long term (Carter and Porell (2011); Inaba et al. 2003).

**Table 3:** Average duration and cost per episode of care, by types of healthcare services and injuries

	Duration (days) ^b^				Cost (2009) ^b^	
Type	Total	ED	In.	SNF	Total	Attributable
Total	17	1	2	5	$9,209	$6,159
Episodes by healthcare services ^a^						
Inpatient or SNF stay	31	1	6	13	$20,126	$13,754
ED visit or ED-related visit	7	1	0	0	$1,134	$508
Non-ED outpatient visit only	8	0	0	0	$745	$471
Episodes by injury ^a^						
Hip fracture	43	1	8	21	$29,939	$23,152
Non-hip fracture	20	1	2	4	$6,843	$4,361
Non-fracture injury	6	1	1	0	$3,171	$1,104

^a^ These rows are hierarchical and mutually exclusive, with highest level of care experienced in the category coming first

^b^ Duration and cost of episodes are conditional on the type of episode

Altogether, we found that the results confirm that the algorithm for identifying episodes of care for fall-related injuries is functioning as intended. When we decomposed an episode, we found that the details present a realistic and coherent story of fall-related injuries and healthcare services. Variation of episode characteristics across medical groups supported the use of a complex algorithm approach, and descriptive statistics on the proportion, duration, and cost of episodes by healthcare services and injuries verified that our results are consistent with other studies. Moreover, robustness checks, presented in Additional file 1: Appendix D, showed that our results are not dramatically sensitive to the assumptions used for the algorithm.

## Conclusion

Given the financial and time limitations involved in using traditional methods (i.e., patient self-report, medical record review) to identify fall-related injuries and associated costs, practical methods that are cost-efficient and easier to implement are becoming more important. Consequently, we developed a practical approach that employs a comprehensive algorithm to identify episodes of care for fall-related injuries and related costs. Overall, this approach attempts to create a more flexible, generic approach to identifying fall-related injuries and costs that can be applied to existing data describing mixed samples of Medicare patients. This algorithm can be used to analyze claims datasets from both FFS Medicare and MA health plans, which often display wide variation in data format and availability. (Fall-related studies can also save time and money by selectively using Medicare datasets, as shown in Additional file 1: Appendix E.) Also, this algorithm uses four steps to identify fall-related injuries in different healthcare settings, and can be used for extracting detailed information on fall-related injuries and examining associated costs. In particular, using this algorithm for estimating fall-related healthcare services expenditure may lead to reduced measurement bias and improved understanding of fall-related costs. Specifically, it allows for further and detailed analysis of the episodes of care for falls and fall-related injuries. For example, we can examine episodes of care and compare the costs and practices across regions and healthcare providers. We can also dissect the episode and examine the frequency and costs of specific types of injuries (e.g., contusions and lacerations). And while studies are often limited in data and only look into acute care, studies that use this algorithm will also be able to examine the long-term effect of falls and fall-related injuries and their association with other health conditions (Roudsari et al. 2005; Sattin et al. 1990). Future studies can also incorporate ISS if measuring severity is of interest, especially since it is a common practice to use the ISS for measuring severity of injuries in trauma studies (Cryer 2006).

Our approach has several limitations. First, while the approach used here was motivated by the need to identify fall-related injuries where actual tracking of falls was infeasible, one limitation of using the Medicare claims data is that it is dependent upon accurate and consistent coding of information by healthcare providers (Jacobsen et al. 1992; Roudsari et al. 2005; Taylor et al. 2011). Thus, inaccurate information may lead to underestimation or overestimation of episodes, episode duration, and cost of falls and fall-related injuries. Second, the use of E codes in the algorithm could have driven some of the observed variation in outcomes such as non-fracture injury counts, as discussed earlier in Table 1, since E-coding varies by hospitals and EDs as well as by states. Thus, an important area for future research is the impact of local coding practice on fall-related injury ascertainment. Third, due to the limitations of our data source, we did not consider whether a specific fall or fall-related injury was the inciting cause of a new episode or the consequence of another acute illness. Future work should address this concern by simultaneous review of claims and electronic health record data. Distinguishing the various types of fall episodes would strengthen the ability to focus on falls that are likely to respond to evidence-based interventions as opposed to those where the fall is a manifestation of another acute illness (e.g., infection, stroke). Fourth, we were unable to compare the algorithm’s results with actual medical records of falls. Fifth, we did not use ICD-9-CM V codes to identify ongoing fall-related costs when calculating attributable cost, which might have additionally captured small amounts of cost that could have been missed. And sixth, CMS switched to using ICD-10 codes as of October 2015. However, this algorithm will still be a useful resource for future researchers investigating fall-related injuries for the following reasons. First, it will still be relevant and useful for analyzing data collected prior to the switch from ICD-9-CM to ICD-10. Moreover, this algorithm can be generally applied and widely used with modification and update of the diagnosis codes.

Consequently, recommendations for future research and algorithm improvements are as follows: (1) updating diagnosis codes from ICD-9-CM to ICD-10 codes, (2) applying V codes to the algorithm to improve accuracy of measuring attributable cost, (3) validating the algorithm using data with medical records or patient self-reports to identify actual falls, (4) examining the impact of local coding practice on fall-related outcomes, and (5) examining duration and cost of episodes of care for fall-related injuries by type of injury, time period, and type of healthcare services.

## Additional file

Additional file 1:
**Includes relevant information that are labeled as Appendix A-F.** Specifically, Appendix A provides the descriptions of the datasets, data cleaning and combining process, and key variables used in the algorithm. Appendix B describes data-specific approaches used to identify inpatient and SNF setting. Appendix C introduces the types of injuries and associated diagnosis and procedure codes used for identifying episodes of care for fall or fall-related injuries. We then present robustness checks for identifying episodes in Appendix D and how the identification of episodes is affected by the datasets used. Lastly, Appendix F provides robustness check for duration of episodes. (PDF 238 kb)

## Abbreviations

BETOS code: Berenson-Eggers Type of Service code
CMS: Centers for medicare and medicaid services
CPT-4 code: Current Procedural Terminology code
DRG: diagnosis-related group
E code: external cause of injury code
ED: emergency department
FFS: fee-for-service
HCPCS code: Healthcare Common Procedure Coding System code
ICD-9-CM: International Classification of Diseases, Ninth Revision, Clinical Modification
ID: identifier
ISS: Injury severity score
MA: Medicare advantage
MedPAR: Medicare provider analysis and review
ResDAC: Research data assistance center
SNF: skilled nursing facility
