# Supplementary material for: Development of an algorithm to identify fall-related injuries and costs in Medicare data
Source: Inj Epidemiol. 2016 Jan 5;3(1):1. doi: 10.1186/s40621-015-0066-z (PMC4701758; doi:10.1186/s40621-015-0066-z)
Supplement: Additional file 1: — Includes relevant information that are labeled as Appendix A-F. Specifically, Appendix A provides the descriptions of the datasets, data cleaning and combining process, and key variables used in the algorithm. Appendix B describes data-specific approaches used to identify inpatient and SNF setting. Appendix C introduces the types of injuries and associated diagnosis and procedure codes used for identifying episodes of care for fall or fall-related injuries. We then present robustness checks for identifying episodes in Appendix D and how the identification of episodes is affected by the datasets used. Lastly, Appendix F provides robustness check for duration of episodes. (PDF 238 kb) [file 40621_2015_66_MOESM1_ESM.pdf]

Appendix A - Data and variables

Medicare FFS datasets included Medicare provider analysis and review (MedPAR), outpatient, hospice, home health agency, durable medical equipment, and carrier files for patient claims and line items. The term "line item" refers to uniquely identified records of information including revenue center codes, Healthcare Common Procedure Coding System (HCPCS) codes, and payment amounts from Medicare to healthcare services providers and suppliers (Centers for Medicare & Medicaid Services). One or more line items comprise a claim; for each claim we extracted aggregated information such as service dates and ICD-9-CM diagnosis codes. We also received comparable MA datasets from 5 health plans in 2 medical groups. Major differences between the FFS Medicare and MA datasets include generally less reported diagnosis and procedure information, and absence of E codes and diagnosis-related groups (DRGs) in the MA datasets. Thus, our algorithm attempts to use information that is common across each dataset. We allow for E codes because this is the single most specific set of information identifying falls that is routinely collected and reported in these types of data. As a result, the key information that we received for all datasets and that we used to identify fall-related injuries and fractures included ICD-9-CM diagnosis codes (including E codes), HCPCS procedure codes, service dates, place of service codes, and revenue codes.

Because of the wide variation in data availability and format, we reviewed each MA dataset to make sure duplicate claims were removed and information was preserved in a consistent manner. At each stage, we used all the information that was available to identify the duplicates (e.g., patient ID, charge or payment amount, date, diagnosis code, HCPCS code, place of service, provider ID). First, line items with negative healthcare costs (suggesting a credit to offset the presence of an erroneous claim) were removed along with line items with matching positive costs. As a result, we removed 2.9% (=660/22481) of the line items for MA dataset 2-1. Next, we removed duplicate items that were present within a given health plan. Specifically, 0.4% (=138/36853) of the line items in MA datasets 2-1 and 2-2 were removed. We also removed 1.4% (=124/8964) of the line items in MA dataset 5. Lastly, we compared across health plans and removed 77 duplicate line items among a total of 307527.

Table A.1 shows the variation observed in different datasets. All data sources provided information on the service dates, primary ICD-9-CM diagnosis code, and HCPCS procedure code. Other key information including secondary ICD-9-CM procedure codes, place of service, and revenue codes were variably included. For cost estimation purposes, we collected information on both payment and charge amounts and used payment amounts whenever available. Other variables that were considered but excluded in the construction of the algorithm were DRG and claim identifier (ID) information. Only 3 out of 7 MA datasets (or 3 out of 5 health plans) provided DRG information. The same number of datasets and plans also provided an internally generated ID for claims, but it was not clear how these IDs were defined and constructed so we decided to build our algorithm on dates of service, rather than claim IDs. Since we could not aggregate line item information from MA datasets into claims, line items were used as the basic unit of analysis in the algorithm. The only exception was the MedPAR

dataset, where one record represented one claim in a given inpatient or SNF setting. Consequently, we included claims from the MedPAR dataset in the algorithm as well. FFS and some MA datasets (namely, 1, 2-1, and 2-2) included amounts reimbursed to providers, while the rest of the MA datasets only included amounts charged to the beneficiaries.

Table A.1 Variation in Medicare FFS and MA datasets. [a]

**Data source**	**FFS**	**MA datasets** [b]						
**Description of key variables**		**1**	**2-1**	**2-2**	**2-3**	**3**	**4**	**5**
Start and end of service date	Yes	Yes	Yes	Yes	Yes	Yes	Yes	Yes
Primary ICD-9-CM diagnosis code	Yes	Yes	Yes	Yes	Yes	Yes	Yes	Yes
Maximum number of secondary ICD-9-CM diagnosis codes	7-9 [c]	10	0	2	0	3	0	4
HCPCS code [a]	Yes	Yes	Yes	Yes	Yes	Yes	Yes	Yes
Place of service	Yes	Yes	Yes	-	Yes	Yes	Yes	Yes
Revenue code	Yes	Yes	-	Yes	-	Yes	Yes	-
Charge amount	-	-	-	-	Yes	Yes	Yes	Yes
Payment amount	Yes	Yes	Yes	Yes	-	-	-	-
Diagnosis-related groups (DRG)	Yes	Yes	-	-	-	Yes	Yes	-
Internally generated ID for claims	Yes	Yes	-	-	-	Yes	-	Yes
Number of beneficiaries [d]	1774	367	289	243	243	87	148	46

[a] FFS, fee-for service; MA, Medicare Advantage; HCPCS, Healthcare Common Procedure Coding System

[b] Each MA dataset represent patients in a given health plan. One health plan provided 3 separate datasets with line items for healthcare services depending on the setting and provider of the services, which are labeled as MA datasets 2-1, 2-2, and 2-3.

[c] Maximum number of secondary diagnoses is 9 for outpatient dataset and 7 for the rest of FFS datasets.

[d] These numbers are not mutually exclusive since some people switched health plan coverage during the study period.

Appendix B - Approaches used to identify inpatient and SNF setting

Here we describe the case-by-case approaches used for each MA dataset to identify inpatient and SNF settings. Specifically, we used place of service and DRG information for plan dataset 1. Inpatient line items were identified if there were both an inpatient and DRG code, while SNF line items were solely based on the presence of a place of service code for SNF. For plan dataset 5 we used service type variable information, which classified line items into various settings including inpatient and SNF. For plan dataset 4 we used both place of service and care type information. Line items that involved “inpatient” care in a “hospital inpatient facility” were considered as inpatient while line items that involved “SNF” care in a “skilled nursing facility” were considered as SNF. For plan dataset 3, following the guidelines from the plan providers, we used hospital bill and facility type information. Line items commonly required an “inpatient stay” bill class to qualify as inpatient or SNF; then, the two were sorted in accordance with the accompanying facility type indicating “inpatient hospital” or “SNF”. As specified by the data provider, dataset 2-2 included claims for facilities while datasets 2-1 and 2-3 included services in outpatient settings, which was verified by the nature of services billed for and the dates of service. Since dataset 2-2 only provided information to identify an institutional stay (including inpatient hospital and SNF) or outpatient status, we went through all the names of facilities listed in the dataset to identify claims and line items that were for services provided in inpatient hospital settings, as opposed to SNF settings.

Appendix C - Type of injuries and associated diagnosis and procedure codes

ICD-9-CM diagnosis codes were sufficient to identify fall-related incidents in an inpatient or SNF setting. However, outpatient coding is often less reliable than inpatient coding (where specifically trained personnel are responsible for coding claims), which results in greater complexity for understanding care provided in outpatient settings. As a result, we adopted a more detailed approach by using additional CPT-4 codes on repair, imaging, casting, and splinting to confirm the presence of an injury.

“Head injury” ICD-9-CM codes were only examined when they occurred in an inpatient or SNF setting. CPT-4 codes were not specified since head fractures and traumas would almost always involve a severe injury and an inpatient visit, and should be reliably identified by ICD-9-CM codes alone. We examined “hip fracture” ICD-9-CM codes in outpatient settings if they were accompanied by CPT-4 codes representing a relevant repair procedure. Furthermore, for each of the body sites in “other fractures” and “joint dislocation”, we constructed a comprehensive list of repair and imaging codes and we also applied general casting and splinting CPT-4 codes wherever these were clinically appropriate. Lastly, to identify fall-related use of medical care, we used E codes for falls independently of other ICD-9-CM or CPT-4 codes. Table C.1 provides the list of body sites or types of injury identified in the second step of the algorithm, along with associated diagnosis and procedure codes that were used for identification.

Table C.1 ICD-9-CM diagnosis codes and CPT-4 procedure codes used to identify fall-related incidents.

Procedure codes include repair, casting, splinting, and imaging procedures. Repair and imaging codes are specific to each body site or type of injury whereas casting and splinting codes are general codes that are not tied to each site or injury. Casting codes include 29000-29086 and 29305-29450 while splinting codes include 29105-29131 and 29505-29515. These casting and splinting codes are applied wherever mentioned in the column for repair procedure codes below.

Body site or type of injury	Diagnosis	Repair procedure	Imaging procedure
1. Hip Fracture			
a. Hip fracture	820.xx	27230-27248	
2. Other Fracture			
a. Pelvis	808.xx	27193-27194, 27215-27228	
b. Rib	807.0x-807.1x	21800, 21805, 21810	71010, 71015, 71020, 71021, 71022, 71023, 71030, 71034, 71035, 71100, 71101, 71110, 71111, 71250, 71260, 71270, 71275, 71550-71555

c. Clavicle	810.xx	23500-23515	71010, 71015, 71020, 71021, 71022, 71023, 71030, 71034, 71035, 71250, 71260, 71270, 71275, 71550-71555, 73000, 73020, 73030, 73040
d. Humerus	812.xx	23600-23630, 23665-23680, 24500-24587 plus splint	73020, 73030, 73040, 73060, 73200, 73201, 73202, 73206, 73218, 73219, 73220, 73225
e. Radius & ulna	813.xx	24586, 24587, 24620, 24635, 24650-24685, 25500-25609, 25611, 25620, 25650-25652 plus cast or splint	
f. Navicular (scaphoid)	814, 814.0, 814.00, 814.01, 814.09, 814.1, 814.10, 814.11, 814.19	25622-25628 plus cast or splint	
g.1 Hand - metacarpal	815.xx	26600, 26605, 26607, 26608, 26615, 26645, 26650, 26665, 26740, 26746 plus splint	73120-73225
g.2 Hand - phalanges	816.xx	26720, 26725, 26727, 26735, 26740, 26742, 26746, 26750, 26755, 26756, 26765 plus splint	73120-73225
g.3 Hand - multiple fractures	817.xx	any repair or splint code listed above (for metacarpal or phalanges fracture)	73120-73225
h. Femur, tibia, fibula	821.xx, 823.xx	27500-27514, 27530-27540, 27750-27759, 27780-27792, 27824-27828 plus cast or splint	
i. Patella	822.xx	27520, 27524 plus splint	
j. Ankle	824.xx	27760-27769, 27808-27823, 28430-28445	
3. Head Injury			
a. Head fracture	800.xx-804.xx		
b. Head trauma	850.xx-854.xx		
4. Joint Dislocation			
a. Shoulder	831.xx	23650, 23655, 23660 plus splint	71010, 71015, 71020, 71021, 71022, 71023, 71030, 71034, 71035, 71250, 71260, 71270, 71275, 71550-71555, 73020, 73030, 73040

b. Elbow	832.xx	24600, 24605, 24615 plus splint	73070, 73080, 73085, 73200-73206, 73221-73225
c. Wrist	833.xx	25660, 25670, 25671, 25675, 25676, 25690, 25695 plus cast or splint	73100-73115, 73200-73206, 73221-73225
d. Knee	836.xx	27550, 27552, 27556-27558, 27560, 27562, 27566 plus cast or splint	73560-73580, 73700-73706, 73721-73725
5. E Codes for Accidental Fall			
a. Stairs or steps	E880.x		
b. Ladders or scaffolding	E881.x		
c. Building or structure	E882		
d. Hole	E883.x		
e. One level to another	E884.x		
f. Same level - tripping	E885.x		
g. Same level - pushed	E886.x		
h. Other and unspecified	E888.x		

Table C.2 provides the list of disqualifying E codes used in the fourth step of the algorithm. We examined the full list of E codes in the data and selected codes that were seemingly irrelevant to falls or fall-related injuries. This list can be easily expanded to include a longer list of disqualifying E codes as needed.

Table C.2 Disqualifying E codes.

Description of the cause of injury [a]	E codes
Other specified railway accident	E806.x
Motor vehicle traffic accident involving collision with other vehicle or pedestrian	E812.x-E814.x
Accidental poisoning by gas distributed by pipeline	E867.x
surgical operations, surgical procedures, and other procedures causing abnormal patient reaction	E878.x-E879.x
Foreign body accidentally entering other orifice	E915.x
Struck accidentally by falling object	E916.x
Accidents caused by machinery or cutting and piercing instrument or object	E919.x-E920.x
Adverse effects (caused by antibiotics, anti-infectives, etc.)	E930.x-E936.x
Adverse effects (affecting cardiovascular system, gastro-intestinal system, etc.)	E942.x-E949.x
Poisoning by solid and liquid substances, undetermined whether accidentally or purposely inflicted	E980.x

[a] based on version 31 full and abbreviated code titles (Centers for Medicare & Medicaid Services).

Appendix D - Robustness checks for identification of episodes

We conducted three different types of robustness checks to examine how changing the assumptions at various steps of the algorithm affected the final outcomes of identifying episodes of care. Specifically, we examined three different assumptions in the second, third, and fourth steps of the algorithm.

For the first robustness check, we varied the number of diagnosis codes that were used to identify fall-related injuries in the second step of the algorithm and compared the resulting number of episodes of care. We started with using the primary diagnosis code only, and added secondary diagnosis codes one at a time (as they became available in the claims datasets). As shown in Figure D.1, identification of episodes was most sensitive to the number of diagnosis codes used when there were four or fewer diagnosis codes. We find that identification of episodes is less affected as we gain access to an increasing number of diagnosis codes.

Figure D.1 Total number of episodes of care by number of diagnosis codes used.

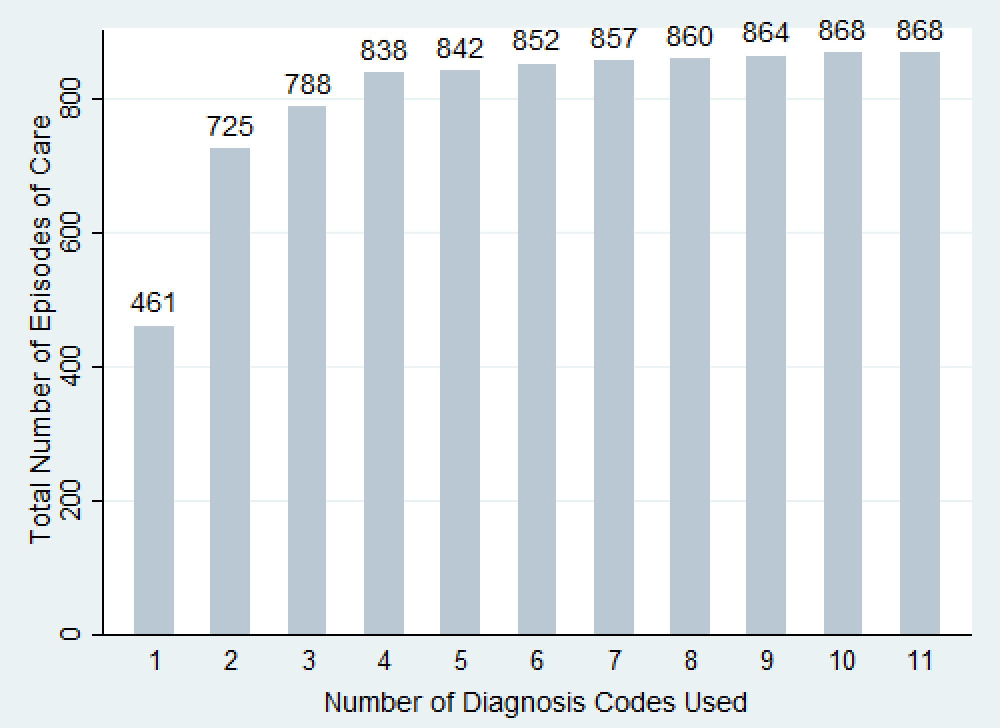

The second robustness check examined one particular type of encounter of fall-related injuries in step 3. In the third step of the algorithm, we used a 10 day window to connect eligible claims and identify the fifth type of encounter of fall-related injuries ("(5) possible outpatient fall-related injury"). Consequently, as a robustness check, we varied the number of days between eligible claims from 1 to 30. As outcomes, we examined the number of episodes that were identified as a "(5) possible outpatient fall-related injury." In Figure D.2, the bars represent the total number of episodes that were possibly outpatient fall-related injuries. We see that there is a positive association between the length of the connection window and the

total number of episodes. With longer connection windows, the algorithm increasingly connects outpatient days with fall-related diagnoses or imaging procedures and identifies them as an encounter for (5) possible outpatient fall-related injury. We also examined how the length of episodes at maximum length and at the 99th percentile were affected and found a drop between 1-2 days (and also between 7-8 days at 99th percentile) and a surge between 13-14 days. Changing the length of the connection window around 10 days had relatively little effect on the number of episodes and length of episodes at maximum length or at 99th percentile.

Figure D.2 Total number of episodes of care by connection window length.

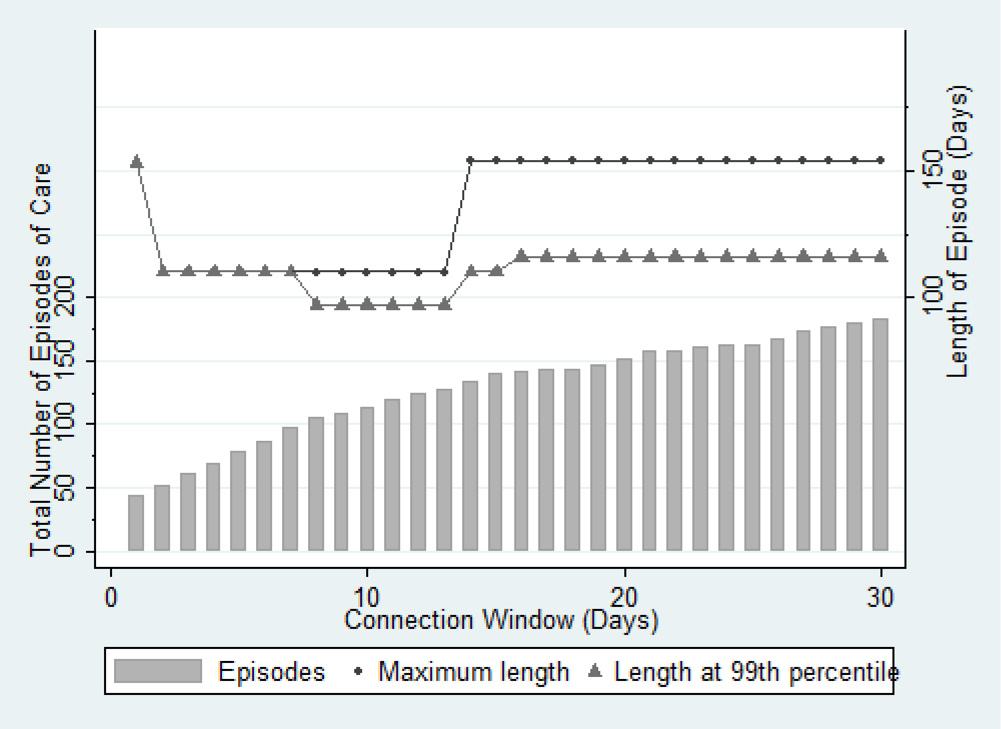

Finally, we looked into the fourth step, where we had used a 30 day window to combine encounters and identify episodes of care. For the third robustness check, we varied the episode window from 2 to 100 days. In Figure D.3, we find that there is a steady yet negative association between the length of the episode window and the total number of episodes. With longer windows, more encounters were combined into episodes and fewer episodes were identified as new episodes. We also examined how the length of episodes at maximum length was affected and found a surge between 56-58 and 66-68 days. Although we did not observe any sudden change in the length of episodes at 99th percentile, we observed that the length of episodes at the 99th percentile increased as the episode window became longer. Again, changing the length of the episode window around 30 days had relatively little effect on the number of episodes and length of episodes at maximum length or at 99th percentile.

Figure D.3 Total number of episodes of care by episode window length.

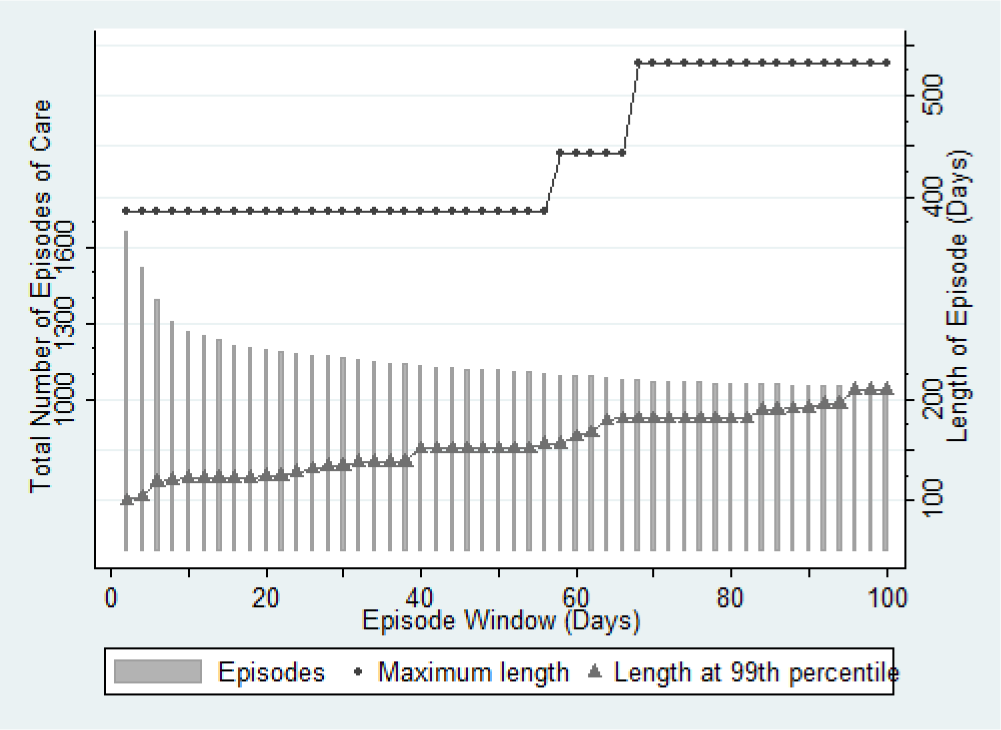

Appendix E - Identification of episodes of care by Medicare FFS datasets

We examined how the identification of episodes of care was affected by the datasets used. We limited the analysis to episodes of care for patients in Medicare FFS and examined how the number of episodes changed as we used five different sets of Medicare FFS datasets. The different sets involved using (1) all Medicare FFS datasets (MedPAR, outpatient, hospice, home health agency, durable medical equipment, and carrier files), (2) MedPAR, outpatient, and carrier files, (3) MedPAR and outpatient files, (4) MedPAR and carrier files, and (5) MedPAR files only. As shown in Table E.1, we can identify most of the episodes by using MedPAR, outpatient, and carrier files. By comparing columns (i) and (ii), we find that little is gained by including additional datasets such as hospice, home health agency, and durable medical equipment files.

Table E.1 Number of episodes of care by Medicare FFS datasets and medical groups.

Medical Groups	(i)<br>All	(ii)<br>M, O, C [a]	(iii)<br>M & O [a]	(iv)<br>M & C [a]	(v)<br>M [a]
A	386	386	175	377	127
B	210	208	109	196	78
C	100	97	47	90	30
D	130	130	88	96	39
E	42	41	27	37	17
Total	868	862	446	796	291

[a] "M", "O", and "C" refer to MedPAR, outpatient, and carrier files, respectively.

Appendix F - Robustness check for duration of episodes

We used ordinary least squares regression models to examine how severity affected the duration of episodes while controlling for health plans and medical groups. We find that injury severity is associated with the duration of episodes even when controlling for the variation in differences in health plans and medical groups. We used type of healthcare service as proxy for injury severity. We classified all episodes into one of the following four mutually exclusive categories, in descending order of severity: episodes with SNF stay, which imply an injury severe enough to require rehabilitation; episodes with inpatient stay (but no SNF stay); episodes with ED visit (but no hospitalization); and episodes with outpatient visits only (but no ED visits). We used the last category as the baseline and examined four different specifications: (1) baseline model, (2) model with a dummy variable for medical group (i.e., whether the patient was covered by MA or FFS), (3) health plan fixed effects, and (4) individual patient fixed effects. We find that episodes with a SNF stay are more likely to have significantly longer duration than episodes with an outpatient visit only. Episodes that involve inpatient stays also show longer but insignificantly increased duration than episodes with outpatient visits only. We also see that episodes that involved patients covered by MA had shorter duration.

Table F.1 Association between the duration of episodes and injury severity

Duration of episode (in days)	(1) Baseline	(2) Coverage	(3) Plan FE	(4) Patient FE
Episodes with SNF stay	46.50***	46.17***	46.09***	45.26***
	(3.338)	(3.330)	(3.341)	(6.793)
Episodes with inpatient stay	2.799	2.272	2.354	0.786
	(3.145)	(3.140)	(3.151)	(6.215)
Episodes with ED visit	-1.028	-1.298	-1.226	-4.113
	(2.938)	(2.930)	(2.937)	(5.853)
Dummy variable for medical group (=1 if MA)		-4.738***	-3.503*	-19.66**
		(1.653)	(2.013)	(9.286)
Constant	7.208***	9.133***	7.752**	16.12***
	(2.723)	(2.797)	(3.058)	(6.222)
Health plan fixed effect	No	No	Yes	No
Patient fixed effect	No	No	No	Yes
Observations	1,162	1,162	1,162	1,162
R-squared	0.295	0.300	0.301	0.250

Standard errors in parentheses

*** p<0.01, ** p<0.05, * p<0.1

References

Centers for Medicare & Medicaid Services. Chapter 1- General Billing Requirements, Medicare Claims Processing Manual. http://www.cms.gov/Regulations-and-Guidance/Guidance/Manuals/Downloads/clm104c01.pdf. Accessed February 2014.

Centers for Medicare & Medicaid Services. ICD-9-CM Diagnosis and Procedure Codes: Abbreviated and Full Code Titles. http://www.cms.gov/Medicare/Coding/ICD9ProviderDiagnosticCodes/codes.html. Accessed February 2014.
